# Adjuvant Denosumab therapy following curettage and external fixator for a giant cell tumor of the distal radius presenting with a pathological fracture: A case report

**DOI:** 10.1016/j.ijscr.2022.107342

**Published:** 2022-06-24

**Authors:** Arulanantham Arulprashanth, Aadil Faleel, Chamikara Palkumbura, Umesh Jayarajah, Rukshan Sooriyarachchi

**Affiliations:** Department of Orthopaedics and Trauma, National Hospital of Sri Lanka, Colombo, Sri Lanka

**Keywords:** Denosumab, External fixator, Giant cell tumor, Distal radius fracture, Pathological fracture, Case report

## Abstract

**Introduction and importance:**

Denosumab is used as a neoadjuvant therapy for giant cell tumours (GCT) prior to surgery to improve surgical clearance and reduce the rate of recurrence. However, the use of denosumab as adjuvant therapy following an external fixator for GCT of the distal radius has not been commonly described. We describe the use of adjuvant denosumab following curettage and external fixation in a patient with GCT of the distal radius presenting with a pathological fracture.

**Case presentation:**

A 23-year-old male presented with a right distal radius fracture. Imaging was suggestive of a Campanacci grade 3 GCT at the distal radius with a pathological fracture. His chest X-ray was normal. He was managed with a dorsal open distal radius curettage and stabilization of the fracture with an external minifixator. Histology confirmed a GCT and adjuvant denosumab therapy was given. The response was satisfactory and the external fixator was removed at 5 months. At 42 months post-treatment, he had satisfactory function with no evidence of recurrence.

**Clinical discussion:**

The extensive involvement of the distal radius and local invasion precluded the use of internal fixation after thorough curettage. Therefore, an external minifixator was applied to stabilize the fracture and started on denosumab following oncology opinion.

**Conclusion:**

External fixation and adjuvant denosumab may be considered as an option in patients who are not suitable for internal fixation. However, cohort studies with long term follow up is necessary before it can be recommended in routine practice.

## Highlights

• External fixation and adjuvant denosumab was effective in our patient with pathological distal radius fracture due to GCT.
• Pre-operative assessment with X-rays and MRI is paramount in planning the surgery.
• However, cohort studies with long term follow up is necessary before routine practice.

## 1 Introduction

Giant cell tumor (GCT) is a bone neoplasm that grows from clusters of neoplastic, mononuclear cells amongst uniformly distributed large osteoclast-like giant cells. It is generally benign in nature with a tendency for local infiltration and rarely metastasize to lungs [1]. Furthermore, it has a recurrence rate of 15–45 % [2]. This osteolytic lesion results from activated osteoclast-mediated bone resorption via the RANK/RANKL pathway [3]. Distal radius is the third commonest site for GCT following knee joint and proximal humerus [4]. Surgery is the main stay of treatment however, the location of the tumor may preclude radical resection due to the functional demand of the involved site.

Denosumab is used as a neoadjuvant therapy for GCT prior to surgery to improve surgical clearance and reduce the rate of recurrence. However, the use of denosumab as adjuvant therapy following an external fixator for GCT of the distal radius has not been described. We describe the use of adjuvant denosumab following curettage and external fixation in a 23-year-old male patient with a Campanacci grade 3 GCT of the distal radius presenting after 3 months following a pathological fracture. The work has been reported based on the SCARE 2020 criteria [5].

## 2 Case presentation

**Fig. 1:**
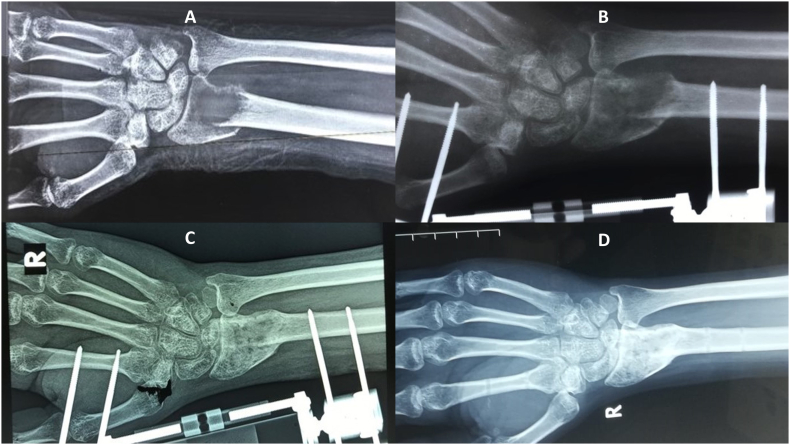
A: R-ray film showing an osteolytic lesion at the distal radius with a pathological fracture; B: after application of an external fixator; C: 3 months after surgery; D: 6 months post-treatment.

**Fig. 2:**
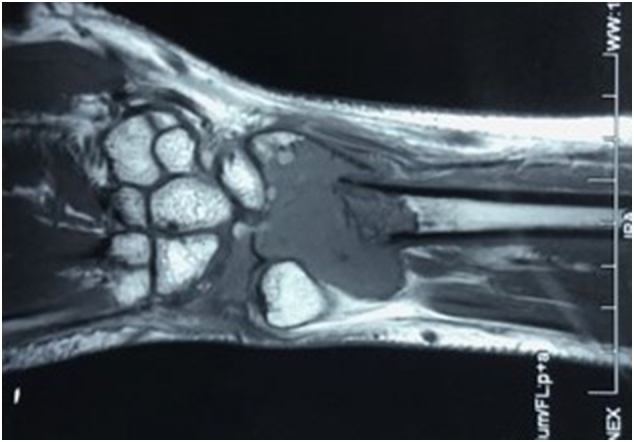
MRI scan of the distal radius showing a Campanacci grade 3 GCT at the distal radius with a pathological fracture.

A previously healthy, 23-year-old, right hand dominant Sri Lankan male presented with pain along the radial side of the right wrist for 3 months following a fall on the outstretched hand. He had no significant drug or allergy history, family history or psychosocial history. Clinical examination was unremarkable except mild swelling and tenderness over the distal radius. X-ray films showed an osteolytic lesion at the distal radius with a pathological fracture (Fig. 1). Magnetic resonance imaging (MRI) showed a probable Campanacci grade 3 GCT at the distal radius with a pathological fracture. The lesion was expansile with extension into the pronator quadratus and showed intense heterogeneous enhancement following intravenous contrast (Fig. 2). His chest X-ray was normal.

**Fig. 3:**
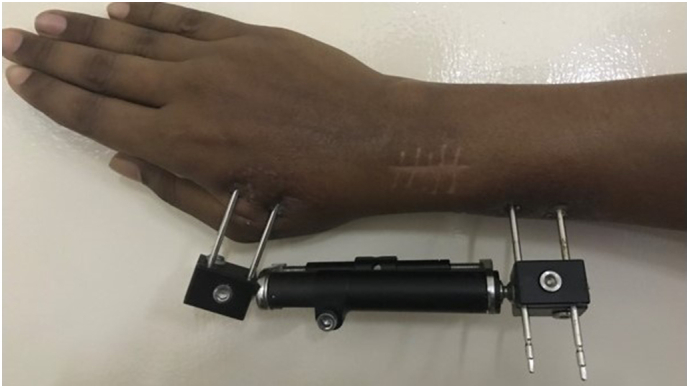
External fixator applied to the distal forearm.

**Fig. 4:**
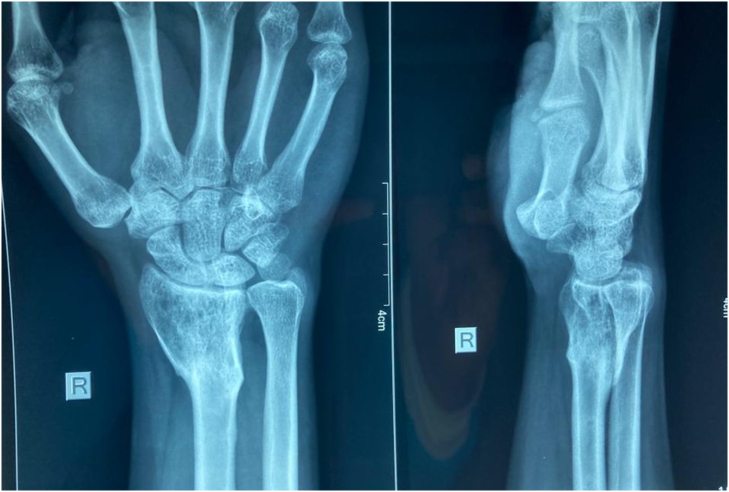
X-rays at 42 months of follow up.

He was managed with a dorsal open distal radius curettage and stabilization of the fracture with an external minifixator. An articulating external fixator was used to allow early range of motion (Fig. 3). The surgery was performed by a senior orthopedic surgeon in a tertiary care hospital. Histology confirmed the diagnosis of a GCT. He was referred to the oncologist and was started on denosumab 120 mg every 4 weekly for a total duration of 5 months as an adjuvant therapy. The dental status was checked and there was no evidence of osteonecrosis. There was a good response to treatment and the external fixator was removed after clinical and radiological confirmation of union at 5 months (Fig. 1). He was started on hand physiotherapy and occupational therapy. At 42 months post-treatment, he had satisfactory hand function with no evidence of recurrence (Fig. 4). His wrist flexion and extension was 60° while radial and ulna deviation were 15 and 20° respectively. He underwent an MRI at 1 year after completion of treatment followed by serial 6 monthly X-rays which did not reveal any recurrence. He is currently on regular surveillance to monitor for recurrence.

## 3 Discussion

GCT of the distal radius is a benign bone tumor however, higher incidence of local invasion and recurrence rate has been reported. Preservation of structural and functional integrity while achieving adequate surgical resection is essential in the management. Therefore, in patients with GCT of the wrist, optimized balance between oncological cure and functional outcome should be achieved [6]. Extra cortical involvement and soft tissue expansion is thought to be common which complicates primary resection of the tumor. In high grade tumours, despite extensive curettage and bone cementing, local recurrence has been reported as high as 88 % [3].

En bloc resection may achieve better local control however, may compromise adequate reconstruction and long term function. Therefore, extensive resection may not be suitable as a standard method of treatment for GCT of the distal radius [6]. Furthermore, some form of reconstruction is necessary after resection of the primary tumor. There are several options available for reconstruction such as osteoarticular allograft, allograft arthrodesis and vascularized or non-vascularized fibular autograft with or without arthrodesis [7]. In cases of pathological factures, some form of fixation is necessary for stabilization. Successful treatment with open reduction and internal fixation has been described in few cases [8], [9]. However due to its rarity, there are no long term data on reconstruction techniques and outcomes of pathological fractures of the distal radius with GCT including sufficient number of patients.

Denosumab, a monoclonal antibody was approved for the treatment of unresectable GCT or where surgical resection is likely to result in severe morbidity [10]. A systematic review by Jamshidi K et al., elaborated the use of denosumab a as neoadjuvant therapy in grade 2 and 3 lesions in order to improve resectability [2]. Recently studies have shown benefits of denosumab as neoadjuvant therapy for GCT in terms of pain reduction and tumor suppression. However, the use of denosumab in patients with GCT in the distal radius is controversial [6]. That is because recent studies have shown that, although it helps in tumor regression, it had no benefits of improving recurrence free survival [2], [6]. Cell culture studies have shown that denosumab was able to clear the giant cells, however, the effect on neoplastic stromal cells were minimal and were continuously proliferating [6]. However, our patient was recurrence free after 42 months of treatment completion. Overall experience of the patient was satisfactory with good functional outcomes.

Our patient presented late with a pathological fracture of the distal radius with GCT. The extensive involvement of the distal radius and local invasion precluded the use of internal fixation after thorough curettage. Therefore, we used an external minifixator to stabilize the fracture and started on denosumab following oncology opinion. Although there are reports of successful use of internal fixation, the successful use of external fixator is not commonly described [8], [9]. Our case report suggests the possibility of the usage of external fixator and adjuvant denosumab for selected patients with GCT who are not suitable for internal fixation. The external fixator was removed after satisfactory union was achieved. At 42 months of follow-up, there was no evidence of recurrence and the functional outcome was satisfactory. However, the patient requires close long term surveillance to monitor for recurrence.

## 4 Conclusion

We described the use of adjuvant denosumab following curettage and external fixator for a late presentation of pathological fracture of the distal radius with GCT. The extensive involvement of the distal radius and local invasion precluded the use of internal fixation after thorough curettage. External fixation with adjuvant therapy may be considered an option in patients who require extensive curettage and who are not suitable for internal fixation. However, cohort studies with long term follow up is necessary before it can be recommended in routine practice.

## Abbreviations

GCT: giant cell tumor
MRI: magnetic resonance imaging

## Sources of funding

None declared.

## Ethical approval

Our institution does not require ethical clearance for case reports.

## Consent

Written informed consent was obtained from the patient for publication of this case report and accompanying images. A copy of the written consent is available for review by the Editor-in-Chief of this journal on request.

## Author contributions

Author AA, CP, AF, UJ and RS contributed to collection of information and writing of the manuscript. Authors UJ and RS contributed to the final approval of the manuscript. All authors read and approved the final version for publication.

## Research registration

Not applicable.

## Guarantor

Rukshan Sooriyarachchi.

## Declaration of competing interest

The authors declare that they have no competing interests.
